# A methodology for examining the association between plasma volume and micronutrient biomarker mass and concentration in healthy eumenorrheic women

**DOI:** 10.7717/peerj.10535

**Published:** 2020-12-21

**Authors:** Sixtus Aguree, Alison D. Gernand

**Affiliations:** 1 Department of Food Science and Human Nutrition, Iowa State University, Ames, IA, United States of America; 2 Department of Nutritional Sciences, The Pennsylvania State University, University Park, PA, United States of America

**Keywords:** Plasma volume, Menstrual cycle, Ovarian cycle, Nutritional biomarker, Indocyanine green, Methodology for micronutrient, Hydration

## Abstract

**Background:**

Accurate estimation and interpretation of nutritional biomarker concentrations are important in nutritional research, clinical care, and public health surveillance. Plasma volume (PV) may affect the interpretation of plasma biomarkers but is rarely measured. We aimed to examine the association between plasma volume (PV) and micronutrient biomarker concentrations and mass as part of pilot work to develop methods.

**Methods:**

Nine healthy women with regular menstrual cycles provided fasting blood samples to measure micronutrient biomarkers. Indocyanine green was injected, and five timed blood draws were taken from 2 to 5 min to measure PV. Visits were scheduled around menstrual cycle day 2. Retinol, 25-hydroxyvitamin D, riboflavin, alpha-tocopherol, zinc, copper, magnesium, manganese, cobalt, iron, and ferritin concentrations were measured in serum. Total circulating micronutrient biomarker mass was calculated from PV and concentration.

**Results:**

The mean PV was 2067 ±  470 mL. PV correlated positively with concentration of iron (*r* = 0.87, *P* = 0.005); other correlations were weaker with *p* > 0.05. PV and total mass of retinol (*r* = 0.90), 25(OH)D (*r* = 0.75), zinc (*r* = 0.88), copper (*r* = 0.83), magnesium (*r* = 0.93), manganese (*r* = 0.72), and iron (*r* = 0.92) were strongly correlated (all* p* < 0.05). PV was positively correlated with circulating micronutrient mass for most biomarkers, implying that concentrations are maintained at different volumes of plasma. Larger studies are needed to further examine these relationships.

**Conclusion:**

Though there appear to be some association between micronutrient biomarker mass and plasma volume, we are unable to draw a firm conclusion about any relationship from these results because of the small sample size. We consider these findings as a preliminary analysis to establish methods for future studies.

## Introduction

Micronutrient status is commonly assessed by measuring circulating serum (or plasma) concentration of specific biomarkers related to the vitamin or mineral. Biomarker concentrations are used by health agencies and professional societies to set different cutoff values to define deficiency (Hess et al., 2007; IOM, 2001; WHO, 2004; WHO, 2009; WHO, 2011). Researchers and public officials use these cutoffs to examine the prevalence of nutritional deficiencies in a population and decide whether public health interventions are needed. For instance, serum zinc concentration is the standard biomarker for identifying zinc status in population studies, and values below 70 µg/dL are interpreted as deficient (Hess et al., 2007; IOM, 2001).

Physiologically, plasma volume (PV) could influence micronutrient biomarker concentrations because biomarkers are transported in plasma. For instance, during pregnancy, the rise in PV corresponds with a parallel increase in copper and a significant fall in zinc (Tuttle et al., 1985), folate (Hall, Pirani & Campbell, 1976), and hemoglobin (Gibson, 1973; Whittaker, Macphail & Lind, 1996) concentrations. It has also been reported that PV correlates with copper, ceruloplasmin, and zinc mass (Tuttle et al., 1985) during pregnancy. Studies assessing the relationship between micronutrient and plasma volume in non-pregnant women are scarce. Understanding how PV influences micronutrient biomarker concentrations in non-pregnant women will improve our understanding and interpretation of their nutrition status and will provide a strong basis for comparing changes occurring during pregnancy. As in pregnancy, PV (Bernstein, Ziegler & Badger, 2001; Cullinane et al., 1995) and micronutrient concentrations (Chandra, Gupta & Patel, 2017; Ha & Smith, 2003; Kim, Yetley & Calvo, 1993; Michos et al., 2010) may also vary across the menstrual cycle, which could affect how nutritional status is classified. Some studies have chosen to restrict all measurements to the early follicular phase for all study participants (Damron et al., 2004; Donckers et al., 2012; Krabbendam et al., 2009), which at least reduces the effect of menstrual cycle variation—which is estimated at 4–13% change in PV (Bernstein, Ziegler & Badger, 2001; Cullinane et al., 1995; Spaanderman et al., 2000).

Though research on PV has been encouraged, studies in both pregnant and non-pregnant women are scarce (Faupel-Badger et al., 2007; Schisterman, Mumford & Sjaarda, 2014), particularly so in non-pregnant women. This paper intended to measure PV and biomarkers in non-pregnant, healthy women of reproductive age under conditions where menstrual cycle variability in PV is limited by collecting data during a standard time during the menstrual cycle (i.e., the follicular phase). We hypothesized that PV would be associated with some but not all concentrations of nutritional biomarkers. This pilot study was conducted to generate preliminary data on the association between PV and micronutrient biomarkers in non-pregnant, healthy women of reproductive age to set the stage for a larger study across the menstrual cycle.

## Materials & Methods

### Subjects

Nine healthy women with a regular menstrual cycle participated in this pilot study. Inclusion criteria for the study were as follows: (1) age 18–35 y, (2) regular menstrual cycle (26–35 days), (3) general good health (does not have a known, ongoing health condition/medical issue that requires regular monitoring by a doctor) (4) BMI from 18.5 to 29.9 kg/m^2^, (5) nonsmoking, 76) non-pregnant, and (7) if ever pregnant, last pregnancy ended >12 months before enrollment. Exclusion criteria for the study were as follows: (1) known allergy to shellfish or iodine, (2) blood pressure on the day of measurements was low or high (SBP <90 or >140 mmHg and/or DBP <60 or >90 mmHg), (3) current hypertension or previous hypertensive disorder in pregnancy (gestational hypertension or preeclampsia), (4) taking regular physician-prescribed medication(s) for a health condition, (5) trying to conceive, (6) using hormonal birth control (within the past three months), or (7) currently breastfeeding.

### Ethics approval and consent to participate

The protocol was approved by The Office for Research Protections (ORP) at the Pennsylvania State University (STUDY00004051) and conducted in line with the Declaration of Helsinki. All participants provided written informed consent before enrolling in the study.

### Study design

Participants responded to the study advertisements seeking healthy female volunteers in the State College, Pennsylvania area. The participants were pre-screened via telephone. Those eligible were scheduled to visit the Clinical Research Center (CRC), a service unit in The Pennsylvania State University’s Clinical and Translational Science Institute, University Park, PA, where the study was conducted. Participants came to the CRC in the morning after a 12-hour overnight fast. Study visits were scheduled to occur at menstrual cycle day 2 (the second day from the start of menstrual blood flow as reported by the participant) to 5 to remove the effect of menstrual cycle phase variability, if any, on micronutrient concentrations and PV. One participant was measured on cycle day 9 because that was the only day that she was available. Participants completed a brief questionnaire that included socio-demographics, health history, and pregnancy history, and a trained study staff measured their weight, height, and percent body fat (assessed using bioelectric impedance analyzer) using a standard protocol. They also provided a urine sample for a pregnancy test. Participants then rested in a supine position on a bed in preparation for an intravenous (IV) catheter insertion and blood collection.

### Blood collection and PV determination

After 15 min of rest, an IV was inserted in the left or right antecubital vein. We collected participants’ blood into three different tubes; 6 mL EDTA-free evacuated trace element free tubes and 6 mL and 2 mL EDTA-containing evacuated trace element free tubes for serum, plasma, and whole blood, respectively. Detailed methods of our PV protocol based on the indicator-dilution principle are published elsewhere (Aguree & Gernand, 2019). In brief, a bolus dose of indocyanine green (ICG, IC-GREEN, AKORN Inc, Lake Forest, IL, USA) (0.25 mg/kg body weight) was injected into the IV and flushed with saline solution. From 2 to 5 min after injection, blood samples were collected at intervals of ∼45 s into a 3 mL EDTA tube (5 tubes total). Plasma was obtained from the 6 mL (pre-injection) and 3 mL (post-injection) EDTA tubes and used to determine PV within 2 h of blood collection.

### Measurement of micronutrient biomarkers

Whole blood was run for a complete blood count using the Beckman Coulter Ac-T Diff 2 hematology analyzer (Beckman Coulter Inc, Brea, CA, USA) with 3-level quality-control materials (Beckman Coulter, Inc. Brea, CA) within one hour of blood collection. Serum zinc and copper concentrations were measured using flame atomic absorption spectroscopy on an AAnalyst 400 Spectrometer (PerkinElmer, Inc., Waltham, MA, USA). Serum retinol concentration was measured using ultra performance liquid chromatography (ACQUITY UPLC System, Waters Corporation, Milford, MA, USA). We used quality control samples from the National Institute of Standards and Technology (NIST) reference materials (SRM^®^ 1950, Gaithersburg, Maryland, USA) for the analysis of serum retinol, zinc, and copper.

The concentrations of serum magnesium, manganese, cobalt, and iron were analyzed using inductively coupled plasma mass spectrometry (ICP-MS) with collision cell technology (CCT), using a Thermo Fisher Scientific X Series 2 (Thermo Fisher Scientific, Lanham, MD, USA) with an ASX 250 autosampler, in the Laboratory for Isotopes and Metals in the Environment (LIME) at The Pennsylvania State University. The method was adapted from previous studies (Bumoko et al., 2015; Cao et al., 2019; Muñiz et al., 2001). In brief, serum samples were diluted 25-fold with 0.1 N HNO_3_ (trace metal grade, Fisher Scientific) in 15 mL trace mineral-free centrifuge tubes and stored for 24 h before analysis. Control samples (seronorm L-2; Seronorm™ Trace Elements Serum; SERO AS, Billingstad, Norway) were treated, prepared, and analyzed by the same method as the participant samples.

Serum ferritin and soluble serum transferrin receptor (sTfR) were measured using enzyme-linked immunosorbent assay (ELISA) methods (Ramco Laboratories, Inc., Strafford, Texas, USA). We used commercial ELISA kits to measure serum AGP (Kent Laboratories Inc., Bellingham, Washington) and CRP (Kent Laboratories Inc., Bellingham, Washington, USA). A DEQAS (the Vitamin D External Quality Assessment Scheme) certified laboratory—the Analytical Facility for Bioactive Molecules Laboratory at The Hospital for Sick Children in Toronto, Canada—measured serum concentration 25-hydroxyvitamin D, 3-epi-25(OH)D_3_, α-tocopherol, and riboflavin assays for the current study.

The following cutoffs were used: marginal vitamin A deficiency, serum retinol <0.70 µmol/L (Pee & Dary, 2002); vitamin D deficiency, 25-hydroxyvitamin D <30 nmol/L (IOM, 2011); inadequate vitamin E, α-tocopherol <12 µmol/L (IOM, 2000); zinc deficiency, serum zinc concentration <70 µg/dL (Hess et al., 2007; IOM, 2001); copper deficiency, serum copper <10.0 µmol/L (IOM, 2001); and iron-deficiency, serum ferritin concentration <15 µg/L (WHO, 2011); We defined signs of inflammation as CRP >5.0 mg/L or AGP >1.0 mg/L (WHO/CDC, 2007), and sTfR >8.3 mg/L (Grant et al., 2012). Body iron was estimated using the Cook’s equation (Cook, Flowers & Skikne, 2003).

### Statistical analysis

Arithmetic means and standard deviations (mean ± SD), median (IQR), geometric means (95% CIs), and range (minimum, maximum) were reported for biomarker concentrations and mass (see Fig. S1. Q-Q plots and kernel density plots for biomarker concentrations and mass). Because serum ferritin, zinc, and retinol are known to be influenced by inflammation (Stephensen & Gildengorin, 2000; Thurnham, 2014; Thurnham et al., 2010), measured values were corrected for inflammation (CRP and AGP values) using external correction factors (Fiorentino et al., 2018; Thurnham et al., 2003). The total circulating mass for each biomarker was estimated as the product of the PV and biomarker concentration:

Biomarker mass= PV* biomarker concentration. A Spearman correlation coefficient was computed to assess the relationship between PV and each of the micronutrient biomarkers (concentration and mass). Data were analyzed using Stata version 14 (Stata-Corp., College Station, Texas, USA), and statistical significance (a 2-sided test) was determined based on a nominal level of alpha equal to 0.05.

## Results

The mean age (years), BMI, percent body fat (%), and PV were 25.0 ± 4.5 years, 23.5 ± 2.9 kg/m^2^ (two women had BMI of 26.1, and 29.6, the rest were between 20.3 to 24.2 kg/m^2^), 28.6 ± 5.0%, and 2067 ± 470 mL, respectively. Eight women self-identified as white and one as African-American. All women were nulliparous except one. Two women were classified as overweight; the other seven were normal weight. The concentrations of the biomarkers were within normal ranges (Table 1). CRP was undetectable for all subjects; two participants had elevated AGP concentrations. Summary statistics of parameters from complete blood cell counts were within normal ranges and are reported in Table S1.

**Table 1:** Micronutrient biomarker concentrations and mass in reproductive-age women (*n* = 9).^a^

Biomarker	Mean ± SD	Median (IQR)	GM (95% CI)^b^	Range ^c^
**Concentration of biomarkers**				
Retinol, µg/dL	38.4 ± 6.9	36.7 (33.0, 42.2)	37.9 [33.1, 43.3]	29.3–52.8
25(OH)D _3,_nmol/L	75.7 ± 28.1	79.4 (52.7, 100.1)	70.2 [50.2, 98.3]	29.0–112.6
3-epi-25(OH)D _3,_ nmol/L	3.3 ± 2.5	2.5 (1.9, 4.1)	2.8 [1.7, 4.4]	1.2–9.5
Riboflavin, nmol/L	19.1 ± 7.6	18.0 (13.8, 22.3)	18.1 [13.8, 23.6]	11.3–36.4
*α*-tocopherol, µmol/ L	21.7 ± 6.0	20.2 (16.1, 27.5)	21.0 [17.1, 25.8]	15.3–31.7
Zinc, µg/dL	77.1 ± 12.5	83.0 (64.0, 89.0)	76.2 [66.9, 86.8]	59.0–89.5
Copper, µg/dL	106.6 ± 15.2	102.2 (95.7, 120.5)	105.7 [95.1, 117.5]	88.4–132.2
Magnesium, mg/L	26.6 ± 2.4	27.5 (24.4, 28.5)	26.5 [24.7, 28.5]	22.6–29.9
Manganese, ng/mL	2.6 ± 0.4	2.5 (2.5, 2.8)	2.6 [2.4, 2.9]	2.3–3.5
Cobalt, ng/mL	1.2 ± 0.4	1.1 (0.8, 1.5)	1.1 [0.9, 1.4]	0.8–1.8
Iron, µg/dL	123.4 ± 39.2	120.5 (92.4, 148.2)	118.2 [93.1, 150.1]	76.3–202.4
Ferritin, ng/mL	25.9 ± 16.4	20.9 (9.7, 40.5)	21.1 [12.2, 36.4]	7.9–52.0
sTfR^d^, mg/L	5.2 ± 3.6	3.9 (3.3, 5.0)	4.5 [3.0, 6.8]	3.0–14.0
Hemoglobin^d^, g/dL	12.3 ± 0.7	12.1 (11.8, 12.8)	12.2 [11.7, 12.8]	11.3–13.4
Hematocrit^d^, %	37.1 ± 2.3	37.6 (34.6, 38.7)	37.0 [35.2, 39.0]	34.4–40.5
**Circulating mass of biomarkers**				
Retinol, µg	800.6 ± 287.2	661.6 (636.3, 951.2)	764.5 [604.1, 967.3]	550.8–1468.0
25(OH)D _3,_ µg	64.2 ± 29.8	59.0 (40.9, 94.2)	56.8 [36.8, 87.8]	17.4–106.0
3-epi-25(OH)D3, µg	2.8 ± 2.0	2.3 (1.4, 3.3)	2.2 [1.3, 3.8]	0.7–7.6
Riboflavin, µg	14.5 ± 6.0	11.8 (10.6, 17.7)	13.6 [10.4, 17.8]	10.1–27.2
*α*-tocopherol, mg	18.6 ± 3.5	19.4 (15.7, 21.1)	18.3 [15.8, 21.2]	13.3–24.2
Zinc, mg	1.6 ± 0.5	1.8 (1.1, 2.0)	1.5 [1.2, 2.0]	0.9–2.5
Copper, mg	2.2 ± 0.6	2.1 (1.7, 2.5)	2.1 [1.7, 2.6]	1.5–3.5
Magnesium, mg	55.5 ± 15.6	55.2 (40.8, 68.8)	53.6 [43.0, 66.8]	36.1–79.7
Manganese, µg	5.4 ± 1.2	5.5 (4.4, 6.3)	5.3 [4.5, 6.3]	3.8–7.2
Cobalt, µg	2.4 ± 0.9	2.6 (1.4, 3.0)	2.3 [1.6, 3.1]	1.2–4.0
Iron, mg	2.7 ± 1.4	2.4 (1.4, 3.8)	2.4 [1.6, 3.6]	1.2–5.4
Ferritin, µg	55.7 ± 39.5	45.2 (19.2, 88.2)	42.6 [22.7, 79.8]	12.7–124.8
sTfR^d^ mg	10.2 ± 7.2	7.6 (5.8, 11.5)	8.8 [5.6, 13.9]	4.8–27.1
Hemoglobin^d^, g^e^	365.2 ± 91.2	346.0 (297.1, 404.2)	356.6 [294.8, 431.2]	279.9–558.8

**Notes.**

IQR Interquartile range (lower quartiles, upper quartiles) GM geometric mean 25(OH)D_3_ 25-hydroxyvitamin D_3_; 3-epi-25(OH)D_3_, 3-epi-25-hydroxyvitamin D _3_ sTfR serum soluble transferrin receptor Biomarker mass PV*Biomarker concentration

a All biomarkers were measured in serum, except for hemoglobin which was obtained from whole blood.

b Values are geometric mean (lower 95% CI of geometric mean, upper 95% CI of geometric mean).

c Range (minimum value, maximum value).

d Data available for eight subjects.

e Mass estimated as BV*hemoglobin concentration (where BV, blood volume; PV, plasma volume; hct, hematocrit (%)).

Three women had zinc deficiency, and an equal number were anemic. Only one woman had vitamin D insufficiency, but none were deficient. None of the participants were deficient in iron (by ferritin), vitamin E, vitamin A or copper. The geometric mean of body iron was 3.50 mg/kg (95% CI [1.35–9.08]); one person was iron deficient using body iron (<0 mg/kg).

Overall, the relationships between PV and biomarker mass were stronger than the relationships between PV and biomarker concentrations (Table 2). Correlations between PV and micronutrient biomarker concentrations ranged in absolute value from 0.03 to 0.87, with five negative associations. Only serum iron concentration was statistically significantly correlated with PV. The correlations between biomarker mass and PV ranged from 0.35 to 0.93; all correlations were positive, and nine were statistically significant (retinol, 25(OH)D, α-tocopherol, zinc, copper, magnesium, manganese, iron, and hemoglobin mass).

**Table 2:** Spearmans correlation coefficients of PV with concentration and mass of micronutrient biomarkers in reproductive-age women (*n* = 9).^a^

	Spearman r	
	r	P^c^
**Concentration of biomarkers**		
Retinol, µg/dL	0.03	0.948
25(OH)D3, nmol/L	0.33	0.385
3-epi-25(OH)D3, nmol/L	0.28	0.463
Riboflavin, nmol/L	−0.40	0.291
α-tocopherol, µmol/ L	−0.53	0.148
Zinc, µg/dL	0.59	0.103
Copper, µg/dL	0.03	0.948
Magnesium, mg/L	0.43	0.250
Manganese/mL	−0.08	0.850
Cobalt, ng/mL	0.26	0.502
Iron, µg/dL	0.87	0.005
Ferritin, ng/mL	0.25	0.521
sTfR ^b^, mg/L	0.26	0.536
Hemoglobin^b^, g/dL	−0.11	0.807
Hematocrit^b^, %	−0.04	0.944
**Circulating mass of biomarkers**		
Retinol, µg	0.90	0.002
25(OH)D3, µg	0.75	0.026
3-epi-25(OH)D3, µg	0.53	0.148
Riboflavin, µg	0.62	0.086
*α*-tocopherol, mg	0.35	0.359
Zinc, mg	0.88	0.003
Copper, mg	0.83	0.008
Magnesium, mg	0.93	0.001
Manganese, µg	0.72	0.037
Cobalt, µg	0.53	0.148
Iron, mg	0.92	0.001
Ferritin, µg	0.57	0.121
sTfR^b^, mg	0.62	0.115
Hemoglobin^b^, g^d^	0.90	0.005

**Notes.**

25(OH)D3 25-hydroxyvitamin D3 3-epi-25(OH)D3 3-epi-25-hydroxyvitamin D3 sTfR serum soluble transferrin receptor

Biomarker mass=PV*Biomarker concentration.

a All biomarkers were measured in serum, except for hemoglobin which was obtained from whole blood.

b Data available for eight subjects.

c Statistical significance (a 2-sided test) was determined based on a nominal level of alpha equal to 0.05.

d Mass estimated as BV*hemoglobin concentration (where BV, blood volume; PV, plasma volume; hct, hematocrit (%)).

## Discussion

The purpose of this study was to examine the relationship between PV and micronutrient biomarker concentrations to provide a direction for future large studies. Overall, PV showed a strong positive correlation with the circulating mass of the micronutrients under consideration. But the micronutrient concentrations were not associated with PV. The mean values from the present study are consistent with previously reported values for manganese (Romero et al., 2001; Wang, Du & Zheng, 2008), iron (Næss-Andresen et al., 2019), magnesium (Lowenstein & Stanton, 1986), zinc (Hennigar et al., 2018; Rükgauer, Klein & Kruse-Jarres, 1997), copper (Rükgauer, Klein & Kruse-Jarres, 1997), iron (Dale, Burritt & Zinsmeister, 2002), ferritin (Belza et al., 2005; Milman et al., 2017; Næss-Andresen et al., 2019), retinol (Browne et al., 2008; Gillespie et al., 2004; Stephensen & Gildengorin, 2000; Talwar et al., 2005), and alpha-tocopherol (Ford et al., 2006). Data on circulating plasma mass is limited, and therefore, it is difficult to make comparisons with our study. The mean circulating zinc mass estimated for this study is similar to those reported in the literature (1.5 to 2.5 g) (Brown et al., 2004; Jackson, 1989; Linder, 1991). The mean value of circulating iron mass, 2.7 mg, is consistent with the commonly quoted value of 2–4 mg (Ganz, 2013).These findings show that PV may be associated with the circulating mass of many micronutrient biomarkers but not their concentrations. Specifically, high PV was associated with elevated circulating masses of retinol, 25(OH)D, zinc, copper, magnesium, manganese, iron, and hemoglobin mass.

Physiologically, these findings suggest that the body can adjust to different PV levels, thereby maintaining the micronutrient concentration. At the same time, the amount of circulating mass contracts or expands to help support its function. High PV was associated with a low concentration of some biomarkers (α-tocopherol) while showing a positive association with the biomarker mass. This inverse association suggests a possible dilution effect of PV on the biomarker concentration such that a woman may have normal circulating mass of the biomarker but lower concentration. For biomarkers such as riboflavin and sTfR, the lack of association between biomarker mass and PV could be due to the small sample size, that could be clarified in larger studies.

Given the small sample size, it is unclear if the relationship between PV and biomarkers concentrations (or mass) were distorted by some other factors such as high BMI (overweight) and inflammation. We report statistical testing and *p* values to aid in future work, however we recognize that corrections for multiple testing should be applied if this were not a pilot study.

Though our previous study reported a small non-significant decrease in plasma volume across the menstrual cycle, it is unknown how that may influence micronutrient biomarker concentrations and circulating mass (Aguree et al., 2020). Future studies should be designed (with appropriate statistical power) to clarify whether PV influences micronutrient concentrations and how BMI and inflammation affect these relationships. Despite the challenges with verifying menstrual cycle phases and the variations that may occur in PV throughout the menstrual cycle, this type of work is needed. Future studies should examine factors (e.g., sex hormones concentrations) that contribute to alterations in micronutrient concentration with plasma volume at different time points across the menstrual cycle, and how they are related.

### Limitations

Though carefully designed, the sample size was small; therefore, more extensive studies should explore these relationships across the menstrual cycle.

## Conclusions

This pilot study was designed to develop a method for examining the association between PV and nutritional biomarkers during the early follicular phase of the menstrual cycle. We found that PV was positively correlated with the circulating mass of micronutrient biomarkers in healthy women. PV was not associated with micronutrient concentrations except serum iron. Given the small sample size of the study, we are unable to draw a firm conclusion about any relationship from these results, instead we consider this as a preliminary analysis to establish methods for future studies.

##  Supplemental Information

10.7717/peerj.10535/supp-1 Supplemental Information 1 Q-Q plots and kernel density plots for biomarker concentrations and mass

10.7717/peerj.10535/supp-2 Supplemental Information 2 Contains whole blood complete blood count (CBC) of study participants Complete blood count was measured using the Beckman Coulter Ac-T Diff 2
hematology analyzer (Beckman Coulter Inc, Brea, CA, USA) within one hour of fasted blood sample collection.

10.7717/peerj.10535/supp-3 Supplemental Information 3 Raw data obtained from laboratory analysis used for data analyses and preparation for Tables 1 and 2, and Tables S1 Data were obtained from anthropometry measurement, and laboratory assays including flame atomic absorption spectroscopy on an AAnalyst 400 Spectrometer (PerkinElmer, Inc., Waltham, MA, USA), ultra-performance liquid chromatography (ACQUITY UPLC System, Waters Corporation, Milford, MA, USA), inductively coupled plasma mass spectrometry (ICP-MS), enzyme-linked immunosorbent assay (ELISA) methods (Ramco Laboratories, Inc., Strafford, Texas, USA).

## Additional Information and Declarations

### Data Availability

The following information was supplied regarding data availability: Data are available in the Supplemental Files and Figshare: Aguree, Sixtus (2020): Plasma volume and micronutrient biomarker data. figshare. Dataset. https://doi.org/10.6084/m9.figshare.13249574.v1.
