# Supplementary material for: A methodology for examining the association between plasma volume and micronutrient biomarker mass and concentration in healthy eumenorrheic women
Source: PeerJ. 2020 Dec 21;8:e10535. doi: 10.7717/peerj.10535 (PMC7759127; doi:10.7717/peerj.10535)
Supplement: Supplemental Information 1 [file peerj-08-10535-s001.docx]

Supplemental file 1: **Figure S1. Q-Q plots and kernel density plots for biomarker concentrations and mass**

**Figure S1a**. Q-Q plots for biomarker concentrations

**Figure S1b.** Q-Q plots for biomarker circulating biomarker mass

**Figure S1c**. Kernel density plots for biomarker concentrations

**Figure S1d**. Kernel density plots for biomarker circulating biomarker mass
