# Supplementary material for: A methodology for examining the association between plasma volume and micronutrient biomarker mass and concentration in healthy eumenorrheic women
Source: PeerJ. 2020 Dec 21;8:e10535. doi: 10.7717/peerj.10535 (PMC7759127; doi:10.7717/peerj.10535)
Supplement: Supplemental Information 2 — Complete blood count was measured using the Beckman Coulter Ac-T Diff 2 hematology analyzer (Beckman Coulter Inc, Brea, CA, USA) within one hour of fasted blood sample collection. [file peerj-08-10535-s002.docx]

|  | Mean ± SD | Median (IQR) | GM (95% CI) ^2^ | Range ^3^ |
| --- | --- | --- | --- | --- |
| White Blood Cell Count, x10^3^/uL | 5.8 ± 1.2 | 5.7 (4.8,6.5) | 5.6 [4.8, 6.7] | 4.2–7.9 |
| Lymphocyte Ratio, % | 32.8 ± 9.3 | 31.4 (27.0,35.8) | 31.8 [25.7, 39.4] | 22.5–53.1 |
| Monocyte Ratio, % | 7.2 ± 1.6 | 6.9 (5.8,9.0) | 7.1 [5.9, 8.5] | 5.4–9.6 |
| Granulocyte Ratio, % | 60.0 ± 10.1 | 62.2 (56.8, 67.1) | 59.1 [50.1, 69.7] | 37.4–68.2 |
| Lymphocyte Count, x10^3^/uL | 1.8 ± 0.9 | 1.8 (1.4,2.3) | 1.8 [1.4, 2.2] | 1.3–2.5 |
| Monocyte Count, x10^3^/uL | 0.4 ± 0.1 | 0.4 (0.3,0.5) | 0.4 [0.3, 0.5] | 0.3–0.6 |
| Granulocyte count, x10^3^/uL | 3.5 ± 1.0 | 3.7 (2.6,4.0) | 3.3 [2.5, 4.4] | 1.8– 5.0 |
| Red Blood Cell Count, x10^6^/uL | 4.1 ± 0.3 | 4.2 (3.9,4.4) | 4.1 [3.9, 4.4] | 3.8– 4.5 |
| Mean Corpuscular Volume, fL | 89.6 ± 2.8 | 91.0 (86.7, 91.8) | 89.6 [87.2, 92.0] | 84.9–92.0 |
| Mean Corpuscular Hemoglobin, pg | 29.6 ± 1.3 | 29.9 (28.7, 30.3) | 29.6 [28.5, 30.7] | 27.2–31.2 |
| Mean Corpuscular Hemoglobin Concentration, g/dL | 33.0 ± 0.8 | 33.0 (32.3,33.8) | 33.0 [32.4, 33.7] | 32.0–34.3 |
| Red Blood Cell Distribution Width, % | 12.9 ± 0.7 | 12.9 (12.3,13.7) | 12.9 [12.3, 13.5] | 12.1–14.0 |
| Platelet Count, x10^3^/uL | 205.0 ± 43.7 | 197.3 (176.6, 231.6) | 201.3 [170.1, 238.1] | 152.0–292.5 |
| Mean Platelet Volume, /fL | 8.8 ± 0.7 | 8.7 (8.4, 8.8) | 8.8 [8.3, 9.3] | 8.4–10.4 |
| Plateletcrit, % | 0.180 ± 0.035 | 0.177 (0.155,0.203) | 0.177 [0.152, 0.208] | 0.133– 0.245 |
| Platelet Distribution Width | 15.4 ± 0.3 | 15.4 (15.3, 15.7) | 15.4 [15.2, 15.7] | 15.0– 15.9 |
